# Pneumocystis jirovecii with high probability detected in bronchoalveolar lavage fluid of chemotherapy-related interstitial pneumonia in patients with lymphoma using metagenomic next-generation sequencing technology

**DOI:** 10.1186/s13027-023-00556-1

**Published:** 2023-12-06

**Authors:** Dian Jin, Jing Le, Qianqian Yang, Qianqian Cai, Hui Dai, Liufei Luo, Jiaqi Tong, Wenxiu Shu

**Affiliations:** Department of Hematology, Ningbo Medical Treatment Center Li Huili Hospital, No.1111, Jiangnan road, Ningbo, 315010 China

**Keywords:** Metagenomic next-generation sequencing, Interstitial pneumonia, Lymphoma, Chemotherapy, Pathogens, Pneumocystis jiroyecii

## Abstract

**Background:**

Previous studies achieved low microbial detection rates in lymphoma patients with interstitial pneumonia (IP) after chemotherapy. However, the metagenomic next-generation sequencing (mNGS) is a comprehensive approach that is expected to improve the pathogen identification rate. Thus far, reports on the use of mNGS in lymphoma patients with chemotherapy-related IP remain scarce. In this study, we summarized the microbial detection outcomes of lymphoma patients with chemotherapy-related IP through mNGS testing of bronchoalveolar lavage fluid (BALF).

**Methods:**

Fifteen lymphoma patients with chemotherapy-related IP were tested for traditional laboratory microbiology, along with the mNGS of BALF. Then, the results of mNGS and traditional laboratory microbiology were compared.

**Results:**

Of the 15 enrolled patients, 11 received rituximab and 8 were administered doxorubicin hydrochloride liposome. The overall microbial yield was 93.3% (14/15) for mNGS versus 13.3% (2/15) for traditional culture methods (P ≤ 0.05). The most frequently detected pathogens were *Pneumocystis jirovecii* (12/15, 80%), Cytomegalovirus (4/15, 26.7%), and Epstein-Barr virus (3/15, 20%). Mixed infections were detected in 10 cases. Five patients recovered after the treatment with antibiotics alone without glucocorticoids.

**Conclusion:**

Our findings obtained through mNGS testing of BALF suggested a high microbial detection rate in lymphoma patients with IP after chemotherapy. Notably, there was an especially high detection rate of *Pneumocystis jirovecii*. The application of mNGS in patients with chemotherapy-related IP was more sensitive.

**Supplementary Information:**

The online version contains supplementary material available at 10.1186/s13027-023-00556-1.

## Introduction

Interstitial pneumonia (IP) is a severe adverse effect of chemotherapy in lymphoma patients. It may result in fever, dyspnea, respiratory failure, and death [1]. Additionally, patients who experience IP have more treatment delays and more frequent premature termination of chemotherapy [2]. According to the relevant research, the probability of IP after lymphoma chemotherapy is about 2-30%, and the incidence of IP in patients treated with rituximab-based chemotherapy is significantly higher than those treated with rituximab-free regimens [2–5]. The pathogenesis of chemotherapy-related IP is still unclear. At present, it is considered that the mechanism mainly involves drug allergic reactions and direct drug toxicity that damage endothelial cell membranes, causing diffuse alveolar damage and progressive pulmonary fibrosis [6–8]. However, some researchers have suggested that the occurrence of IP may be due to the increase of opportunistic infections after chemotherapy (especially rituximab-based regimens), as shown by the elevated serum beta-D-glucan (BDG) levels and the detection of certain pathogens (etiological tests are more common for *Pneumocystis jirovecii* (*P. jirovecii*) or fungi) [9, 10]. Many studies have reported that lymphoma patients treated with rituximab regimen are often infected with *P. jirovecii* [11–13]. Furthermore, preventing *P. jirovecii* is effective against *P. jirovecii* infection [14]. However, the pathogen detection rate of chemotherapy-related IP is low when traditional laboratory testing methods are used. Weiping Liu [2] retrospectively analyzed 83 lymphoma patients with IP after chemotherapy. Using traditional laboratory methods, only 6 cases were found to have evidence of pathogenic infection. However, physicians prescribed antibiotics to 53% of those patients with no proof of infection.

Despite less data on chemotherapy-related IP, the role of infection has been well-studied in other types of interstitial lung diseases. Research efforts thus far have shown that viruses are most associated with interstitial lung diseases. The most frequently identified virus appears to be Epstein-Barr virus (EBV), and other viruses including Cytomegalovirus (CMV), and hepatitis C virus [15]. Virus detection mainly relies on polymerase chain reaction (PCR) methods and antibody detection [15]. Bacteria have been less well studied in the area of IP. Richter et al [16] demonstrated positive bronchoalveolar lavage fluid(BALF) cultures for pathogens such as *Streptococcus* species, *Haemophilus* species and *Pseudomonas* species in eight of 22 stable patients with idiopathic pulmonary fibrosis. However, it is uncertain whether the IP caused by bacterial infection or the bacterial infection is secondary to IP. Other bacteria detected in BALF of idiopathic pulmonary fibrosis patients including *Neisseria*, and *Veillonella* [17]. Fungus including *P. jirovecii* and *Aspergillus* were detected as factors associated with IP [18–20]. *P. jirovecii* was usually identified by quantitative real-time PCR or toluidine blue ‘O’ staining on respiratory specimen [21]. Culture and histological-based methods remain central to the diagnosis of *Aspergillus* [22].

Metagenomic next-generation sequencing (mNGS) is a recently developed detection method that is independent of pathogen culture, which can help to provide rapid and objective pathogenic diagnosis. This technology can simultaneously detect known or unknown bacteria, fungi and viruses, and has been proven to significantly improve the pathogen detection rate in systemic infections, artificial joint infections, pleural infections and respiratory infections [23–26]. At present, many studies have confirmed that the analysis of BALF through mNGS can further improve the positive diagnosis rate of pneumonia patients compared with traditional microbiological detection methods [27, 28]. Moreover, mNGS has proven especially suitable for infected patients with negative traditional test results, immunodeficiency patients or critically ill infected patients [29–31]. Therefore, we believe that the application of mNGS to analyze the BALF of patients with IP after chemotherapy is a highly efficient method for the detection of respiratory pathogens.

## Materials and methods

### Ethics statement

The study protocol was approved by the Ethical Review Committee of Ningbo Medical Treatment Center Li Huili Hospital (approval number: KY2022PJ155). Informed consent to publish the information was obtained from the personal patient.

### Case description

**Fig. 1:**
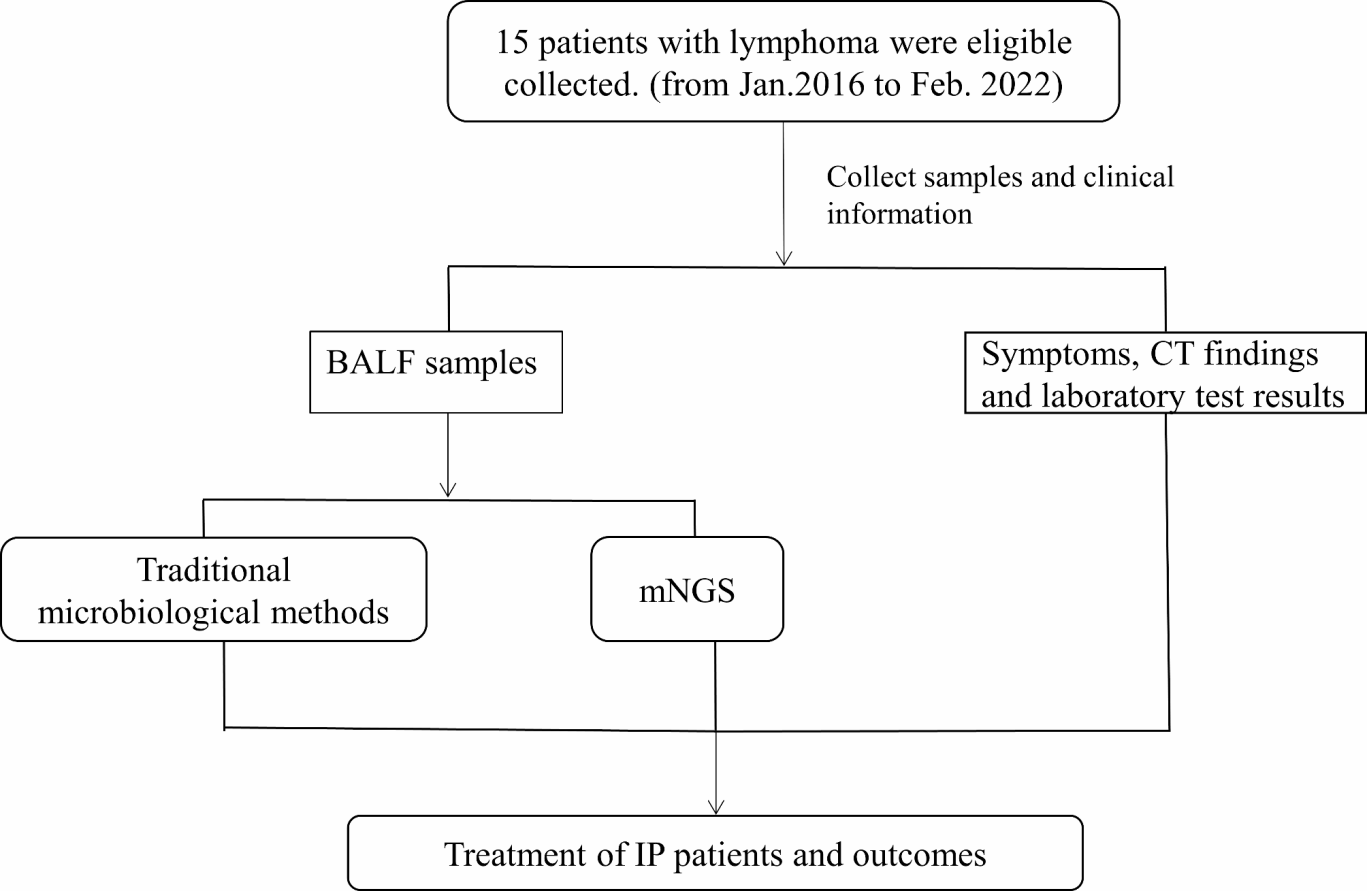
The process of this study

All lymphoma patients who were treated in Ningbo Medical Treatment Centre Li Huili Hospital between January 2016 to February 2022 were retrospectively reviewed. The included study participants were a consecutive series of all patients satisfying the inclusion criteria. The patient eligibility criteria were as follows: (I) ≥ 18 years of age; (II) had a histological diagnosis of lymphoma; (III) received at least one course of chemotherapy; (IV) developed IP after chemotherapy; (V)underwent bronchoscopy and mNGS test of BALF was performed when developed IP. Patients were excluded if they (I)had no BALF culture result; (II) had simultaneous infections of other parts other than the lung, such as blood infection and intestinal infection; (III) had previously received prophylactic anti-*Pneumocystis* treatment with trimethoprim-sulfamethoxazole (TMP-SMX); (IV) had incomplete documentation. IP was diagnosed based on radiologic findings, clinical symptoms, physical examination, laboratory tests, lung function, arterial gas analysis, bronchoalveolar lavage and pathologic results [32, 33]. Arterial gas analysis during the stable phase can show normal results or hypoxemia and respiratory alkalosis [34]. The necessary radiological findings of IP were diffuse pulmonary interstitial infiltration and other manifestations, including traction bronchiectasis, bilateral reticular opacities, loss of lobe volume, and opacity in the lower lungs on computed tomography (CT) scans [32, 33, 35]. The process of this study is shown in Fig. 1.

### BALF collection

The collection of BALF was performed by experienced bronchoscopists according to standard procedures [36]: Prior to the examination, informed consent was obtained, and blood tests, coagulation function, electrocardiogram, and chest CT were used to evaluate the condition. Bronchoalveolar lavage was performed with the fiberoptic bronchoscope in a wedge position within the selected bronchopulmonary segment. Three aliquots of 50 ml normal saline (at room temperature) were instilled through the bronchoscope. After the instillation of each aliquot, instilled saline was retrieved using a negative suction pressure of less than 100 mm Hg. Finally, three aliquots of 20 mL BALF samples were retrieved. The first sample was discarded to avoid contamination, and the other two samples were separated into two aliquots. One was sent for conventional microbial culture in a clinical microbiology laboratory and the other was used for detection using mNGS.

### Traditional microbiological methods

BALF samples from all patients were examined by traditional microbiological methods including smears and cultures for general bacteria and fungi and acid-fast staining for mycobacteria. The following microbiological methods were performed according to the clinician’s discretion: real-time PCR for EBV and CMV from the blood samples, serum BDG assay and serum galactomannan (GM) assay for fungi, serum antibodies for indirect immunofluorescence assay for respiratory syncytial virus, influenza A/B virus, parainfluenza virus, adenovirus, *Legionella pneumophila*, *Mycoplasma pneumoniae* and *Chlamydia pneumoniae*. Unfortunately, due to the limitations of our laboratory, toluidine blue ‘O’ staining on BALF or sputum cannot be performed.

### mNGS of BALF

Total nucleic acid was extracted from 5 ml of BALF. DNA or RNA sequencing libraries were constructed for each patient. An Agilent 2100 instrument was used to assess the quality of the libraries. Then, DNA or RNA libraries were constructed through DNA-fragmentation, end-repair, adapter-ligation, reverse transcription (for RNA) and PCR amplification. High-quality sequencing data were generated by removing low-quality and short (length < 35 bp) reads, followed by computational subtraction of human host sequences mapped to the human reference genome (hg19) using Burrows-Wheeler Alignment. The remaining data by removal of low-complexity reads were classified by simultaneously aligning to four Microbial Genome Databases, consisting of viruses, bacteria, fungi, and parasites. The performers and readers of mNGS company were blinded to patient information, including the symptoms and diagnosis.

We used the following criteria to define clinically significant microbes in this study, which were derived and revised from prior literature [30, 37, 38]. Bacteria (mycobacteria excluded), viruses, fungi, and parasites: A microbe was considered a clinically significant microbe when its relative abundance at the species level was more than 30% and there was supportive literature evidence of its pulmonary pathogenicity. Mycobacterium: Given the low possibility of Mycobacterium contamination and low yield rate, Mycobacterium was considered a clinically significant microbe when the stringently mapped read number at the species level was more than 3.

### Statistical analysis

SPSS 22.0 software was employed for data analysis. The categorical variables were presented as frequency and percentage, and the continuous variables were analyzed using descriptive statistical methods (median and range). The comparison between enumeration data was performed by the chi-square test. P < 0.05 was considered as indicative of statistical significance.

## Results

### Patient characteristics

**Fig. 2:**
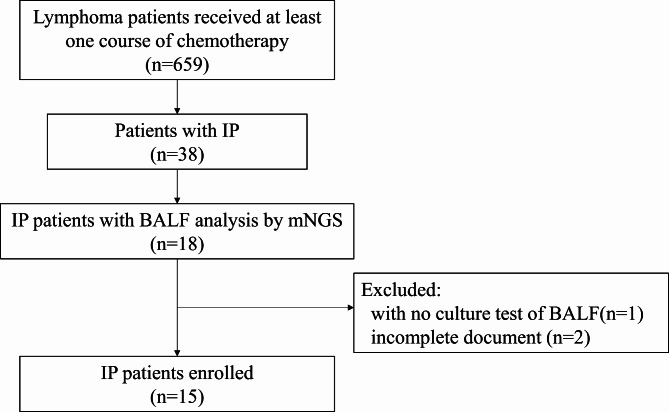
Schematic flow chart of patient selection in this study. IP: interstitial pneumonia; mNGS: metagenomic next-generation sequencing; BALF: bronchoalveolar lavage fluid

During about 6-year study period, 659 lymphoma patients received at least one course of chemotherapy and 38 (5.77%) of these patients developed IP. In total, 15 patients were eligible for this study (Fig. 2). The clinical characteristics of the 15 patients, including sex, age, histology, the international prognostic index (IPI) score at the time of diagnosis of lymphoma, history of smoking, prior lung disease, and the chemotherapies before the onset of IP, were demonstrated in Table 1. The cohort comprised 4 females and 11 males, with a median age of 56 years (range, 30 to 64 years). Most patients (10/15, 66.7%) had a diagnosis of diffuse large B-cell lymphoma (DLBCL), and 66.7% (10/15) patients had IPI score ≥ 2. Few patients presented a history of smoking (4/15, 26.7%) or basic lung disease (1/15, 6.7%). 80% (12/15) of patients received rituximab-based chemotherapies and 53.3% (8/15) of patients received a chemotherapy regimen containing doxorubicin hydrochloride liposome. Only one patient received neither rituximab nor doxorubicin hydrochloride liposome, but they underwent allogeneic hematopoietic stem cell transplantation (allo-HSCT) and used prednisone for anti-rejection. The patients received a median of 4 cycles (1 to 20 cycles) of chemotherapy before they developed IP.

**Table 1:** Basic characteristics of the 15 patients with IP after chemotherapy

Patient ID	Sex	Age (years)	Histology	IPI score	Smoking	Basic lung disease	Chemotherapy	Cycles of chemotherapy before IP
P1	Male	33	DLBCL	2	Yes	No	R-CHOP R-CDOP	4
P2	Male	57	MCL	2	No	Bronchiectasis	R-CHOP CHOP	3
P3	Male	54	HL	1	Yes	No	ABVD	2
P4	Male	49	DLBCL	3	Yes	No	R-ECHOP R-CDOP	4
P5	Female	59	DLBCL	1	No	No	R-CHOP	3
P6	Female	62	DLBCL	1	No	No	CHOP R-CHOP	4
P7	Male	61	FL	2	No	No	R-CHOP	4
P8	Male	54	DLBCL	2	No	No	R-CDOP	5
P9	Female	57	DLBCL	3	No	No	R-CHOP R-CDOP R-ICE	14
P10	Male	64	DLBCL	3	Yes	No	ECHOP R-ECHOP	3
P11	Male	55	DLBCL	1	No	No	R-CDOP R-CHOP	8
P12	Male	30	ENKL	0	No	No	P-GIDE DHAP allo-HSCT Prednisone	20
P13	Female	59	PTCL-NOS	2	No	No	CDOP	1
P14	Male	58	DLBCL	3	No	No	R-EPOCH	2
P15	Male	62	DLBCL	2	No	No	RCDOP	2

*IPI* international prognostic index, *DLBCL* diffuse large B-cell lymphoma, *MCL* mantle cell lymphoma, *HL Hodgkin’s lymphoma*, *FL* follicular lymphoma, *ENKL* extranodal NK/T cell lymphoma, *PTCL-NOS* peripheral T-cell lymphoma-NOS, *R* rituximab, *CHOP* cyclophosphamide, epirubicin, vinorelbine, and dexamethasone/prednisone, *CDOP* cyclophosphamide, doxorubicin hydrochloride liposome, vinorelbine, and dexamethasone/prednison, *ECHOP* etoposide, cyclophosphamide, epirubicin, vinorelbine, and dexamethasone/prednison, *EPOCH* cyclophosphamide, epirubicin, vinorelbine, etoposide, and dexamethasone, *ABVD* doxorubicin hydrochloride liposome, bleomycin, vinorelbine, and dacarbazine, *ICE* ifosfamide, etoposide and cisplatin, *P-GIDE* pegaspargase plus gemcitabine, ifosfamide, dexamethasone, and etoposide, *DHAP* cisplatin, cytarabine and dexamethasone, *allo-HSCT* allogeneic hematopoietic stem cell transplantation

### Symptoms, CT findings and laboratory test results

The patients’ clinical symptoms, characteristic CT findings and laboratory test results were listed in Table 2. Among the 15 patients, only one (P 10) had no obvious symptoms. The most frequent symptoms were fever (14/15, 93.3%) and dyspnea (10/15, 66.7%), and the less common symptoms were cough (2/15,13.3%) and sputum (1/15,6.7%).

**Table 2:** Clinical symptoms, CT findings, laboratory test results and microbial detection results of patients at the time of IP diagnosis

Patient ID	Symptoms	CT findings	Neutrophil count (*10^9^/L)	CRP (mg/L)	BDG and GM assays	Routine laboratory staining and cultures	mNGS outcome	Course of prednisone ≥ 20 mg/d	Antimicrobial therapy	Recovery time
P1	Fever Dyspnea	Diffuse GGO	10	98	Negative	Negative	*Pneumocystis jirovecii*	5 days	TMP-SMX, moxifloxacin	5 days
P2	Fever Dyspnea	Diffuse GGO	8.3	39.3	Negative	Negative	*Campylobacter mucosa* *Pneumocystis jirovecii*	No glucocorticoids were used	TMP-SMX, piperacillin-tazobactam, imipenem/cilastatin	5 days
P3	Fever	Diffuse GGO	0.6	83	Negative	Negative	*Pseudostreptococcus pneumoniae* EBV *Pneumocystis jirovecii*	No glucocorticoids were used	TMP-SMX, piperacillin-tazobactam	20 days
P4	Fever Dyspnea	Diffuse patchy exudation	13.3	62	Negative	Klebsiella pneumoniae	EBV *Candida albicans*	3 months	Caspofungin, linezolid, imipenem/cilastatin	3.5 months
P5	Fever Dyspnea	Diffuse GGO	2.2	10.6	Positive BDG assay (361.9 pg/mL)	Negative	CMV *Pneumocystis jirovecii*	3 months	TMP-SMX, caspofungin, moxifloxacin, voriconazole	1 year
P6	Fever Dyspnea	Diffuse patchy exudation	0.6	61	Negative	Negative	CMV *Pneumocystis jirovecii*	14 days	TMP-SMX, voriconazole, moxifloxacin, teicoplanin	2 months
P7	Fever Dyspnea	Diffuse GGO	2.2	14.1	Negative	Negative	*Pneumocystis jirovecii*	1.5 months	TMP-SMX, caspofungin, voriconazole	5 months
P8	Fever Dyspnea	Diffuse GGO	1.1	277	Positive BDG assay (275.34 pg/mL)	Negative	*Pneumocystis jirovecii*	1 months	TMP-SMX, caspofungin, piperacillin-tazobactam	1.5 months
P9	Fever Cough Dyspnea	Diffuse GGO	2.9	92.4	Negative	Negative	EBV *Pneumocystis jirovecii*	3 months	TMP-SMX, caspofungin, piperacillin-tazobactam	4.3 months
P10	No symptom	Diffuse GGO	2.4	25.2	No data	Negative	Negative	5 days	TMP-SMX	9 days
P11	Fever Dyspnea	Diffuse GGO	20.8	35.8	No data	Negative	*Acinetobacter baumannii* *Pneumocystis jirovecii*	1 month	TMP-SMX, caspofungin, piperacillin-tazobactam	15 days
P12	Fever Cough Sputum	Diffuse GGO	3	34	Positive BDG assay (153.72 pg/mL)	Staphylococcus aureus	*Pseudomonas aeruginosa* *Pneumocystis jirovecii*	No glucocorticoids were used	TMP-SMX, piperacillin-tazobactam	30 days
P13	Fever	Diffuse GGO	10	11.8	Negative	Negative	*Escherichia coli* *Pneumocystis jirovecii* CMV	No glucocorticoids were used	TMP-SMX, caspofungin, piperacillin-tazobactam, ganciclovir	2 months
P14	Fever	Diffuse GGO	8.3	115	Positive BDG assay (129.14 pg/mL)	Negative	*Pneumocystis jirovecii*	No glucocorticoids were used	TMP-SMX, piperacillin-tazobactam	15 days
P15	Fever Dyspnea	Diffuse GGO	0.6	60.6	Negative	Negative	CMV	15 days	TMP-SMX, piperacillin-tazobactam, ganciclovir	1 month

*CT* computed tomography, *GGO* ground-glass opacities, *CRP* C-reactive protein, *BDG* beta-D-glucan, *GM* galactomannan, *mNGS* metagenomic next-generation sequencing, *CMV* Cytomegalovirus, *EBV* Epstein-Barr virus, *TMP-SMX* trimethoprim-sulfamethoxazole

The most common predominant CT patterns were diffuse ground-glass opacities (GGO) in 13 of 15 patients and most of them were symmetric and with no zonal predominance. The other CT manifestations included diffuse patchy exudation and thickening of the interlobular septa. The figures of these CT scans were uploaded as supplement materials.

All patients had elevated C-reactive protein (CRP) (median 64.6 mg/L, range 10.6–227 mg/L), and 40% (6/15) patients had elevated absolute neutrophils (median 2.9*10^9/L, range 0.6–20.8*10^9/L). Serum BDG and GM assays were performed in 13 patients. Four patients had elevated serum BDG assays, and the GM assays were negative in all 13 patients. PCR tests for EBV were performed in 11 patients and were all negative. PCR for CMV was performed in 9 patients, and 1 patient was positive (P 6). Serum antibodies for indirect immunofluorescence assay for respiratory pathogens were performed in 6 patients and were all negative.

### Culture outcomes from BALF

The outcomes of BALF staining and culture were also listed in Table 2. Only two cases (2/15, 13.3%) had positive outcomes, with*Klebsiella pneumoniae* and *Staphylococcus aureus* detected, respectively.

### mNGS results

**Fig. 3:**
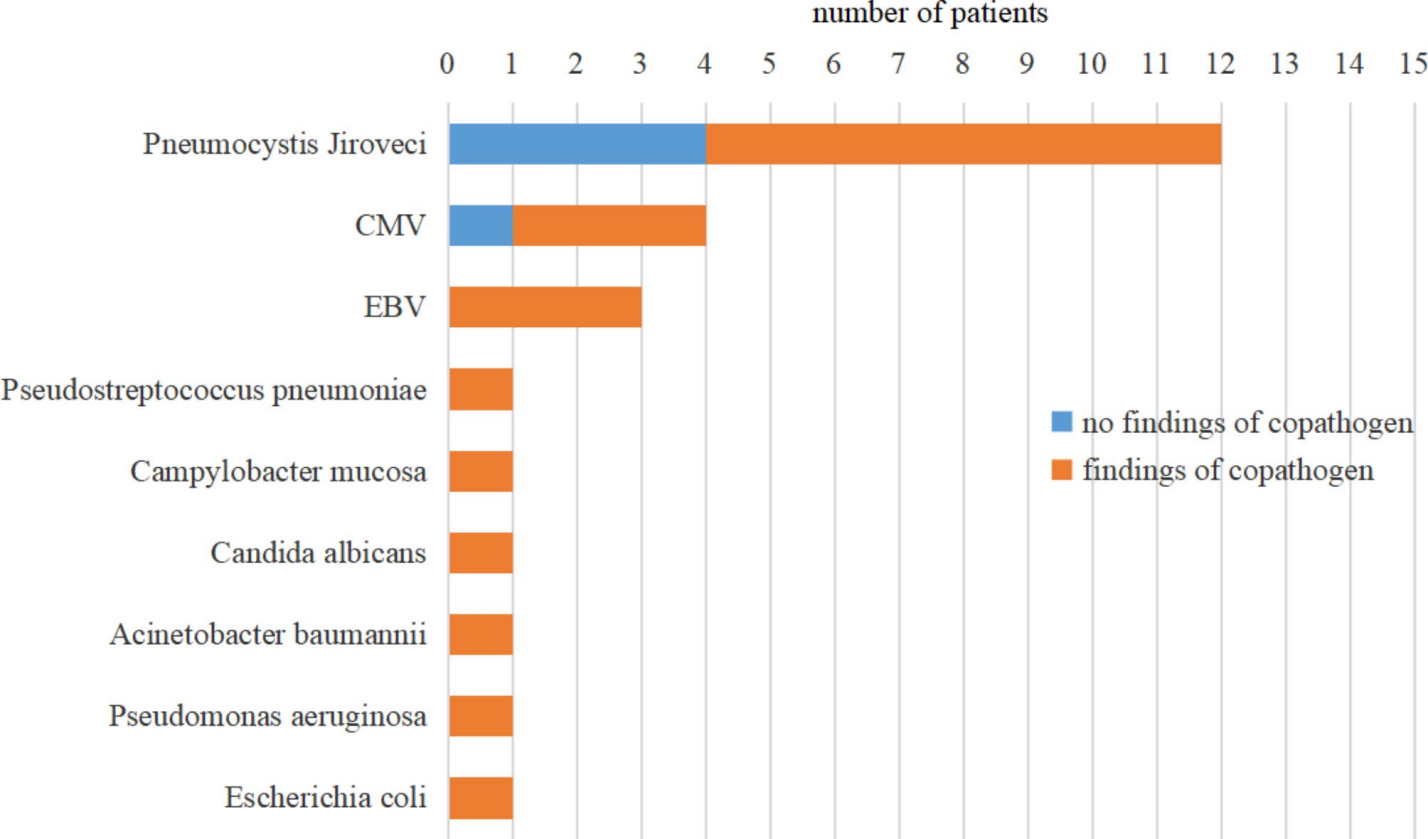
Findings of pathogens of copathogens by mNGS

Pathogens were detected in 14 of 15 (93.3%) patients by mNGS (Table 2). It is worth noting that*P. jirovecii* was detected in 12 patients (12/15, 80%) by this method. Among the patients with *P. jirovecii*, 4 patients had single *P. jirovecii* infection, while the other 8 patients had mixed infections of *P. jirovecii* with other pathogens. CMV and EBV were detected in 4 patients (4/15,26.7%) and 3 patients (3/15, 20%), respectively. Other infrequently detected pathogens were *Campylobacter mucosa*, *Pseudostreptococcus pneumoniae*, *Candida albicans*, *Acinetobacter baumannii*, *Pseudomonas aeruginosa* and *Escherichia coli*. The most common mixed infections were *P. jirovecii* together with CMV or EBV. The identified pathogens and copathogens were listed in Fig. 3.

### Pathogens detected by mNGS relative to conventional microbiologic methods

The overall microbial yield was 93.3% (14 of 15 patients) for mNGS versus 13.3% (2 of 15 patients) for traditional culture methods, with significant difference (Table 2;*P* ≤ 0.05). The pathogens detected by traditional culture methods were different from those detected by mNGS. *Klebsiella pneumoniae* and *Staphylococcus aureus* were respectively identified in P4 and P12 by culture of BALF, but not identified by mNGS. In the 4 patients with positive BDG assay, *P. jirovecii* was detected by mNGS, but no other fungal infection was identified.

### Treatment of IP patients and outcomes

The treatments and outcomes of IP were also presented in Table 2. Treatments for IP included antimicrobial therapies and glucocorticoids. All patients were treated with antimicrobial drugs, and the most commonly used antimicrobial drug was TMP-SMX (14/15). Antibacterial drugs were used in 13 patients, while antifungal drugs were used in 8 patients. Of the 4 CMV-positive patients, 2 were treated with ganciclovir. Most of the enrolled patients (14 of 15) were treated with combinations of multiple antibiotics, and only 1 patient was treated with TMP-SMX alone. Three patients had severe hypoxemia and needed intubation (P5, P8, P15). It is worth noting that 5 patients recovered without the use of therapeutic doses of glucocorticoids (prednisone ≥ 20 mg daily). All of the patients with IP recovered after the applied treatments. Recovery of IP was considered when symptoms and > 75% of interstitial infiltrates disappeared. The recovery times varied between 5 days and 1 year.

## Discussion

The main purpose of this study was to explore the pathogens of lymphoma patients with chemotherapy-related IP using the highly sensitive mNGS method. In previous studies on the etiology of chemotherapy-related IP, conventional microbiological methods are still the mainstream approach and with low detection rates [2, 10]. In this study, using mNGS, we found that the etiological detection rate of patients with chemotherapy-related IP was 93.3%. Moreover, *P. jirovecii* was detected in 80% of patients. The results suggested that detection of pathogens from BALF using mNGS was an extremely sensitive technique in this group of patients, especially for the detection of *P. jirovecii*.

*P. jirovecii* is a frequent opportunistic infection among immunocompromised patients. According to previous literature, *Pneumocystis jirovecii* pneumonia (PJP) occurred in 70–80% of AIDS patients [39]. A national study showed that the largest population associated with *P. jirovecii* were those suffering from underlying hematological malignancy [40]. The diagnosis of PJP include cytochemical or immunofluorescent staining and quantitative real-time PCR assay of respiratory specimen [41–44]. During the last years, the use of PCR has been increasingly investigated because of its high sensitivity. However, the PCR results are often difficult to interpret as they have various gene targets and sensitivities. To be able to differentiate between colonization and infection, standardized tests with data on interpreting low-level positives may be preferred [45]. Recently, mNGS was used for the diagnosis of pneumonia, and *P. jirovecii* was reported to be responsible for 61.2% of confirmed pneumonia in immunocompromised patients [30]. Meanwhile, in the study of pneumonia patients without immunosuppressed state, the detection rate of PJP was low even by mNGS [27, 46]. These results suggest that mNGS is a promising method for the accurate detection of *P. jirovecii* with high sensitivity and specificity. In this study, the enrolled patients suffered severe immunosuppression. It is reasonable to speculate that the *P. jirovecii* detected by mNGS was pathogen causing IP but not merely commensal.

Serum BDG assay and GM assay are helpful for the diagnosis of invasive fungal disease [47]. A meta-analysis showed that the sensitivity and specificity of the BDG assay for the diagnosis of invasive fungal disease were 50-90% and 70-100%, respectively [48]. Another meta-analysis showed the sensitivity and specificity of the BDG assay for invasive fungal disease were 76% and 85% respectively [49]. The BDG assay can also be used to diagnose PJP. Meta-analysis showed that the sensitivity and specificity of serum BDG assay for the diagnosis of PJP were 85% and 73% respectively in patients without human immunodeficiency virus infection [50]. In our study, 4 of 13 patients had elevated serum BDG assay, and *P. jirovecii* was detected by mNGS but no other fungal infection was identified in the 4 patients. This result suggests that BDG assay has good specificity in the detection of *P. jirovecii* in chemotherapy-related IP.

*P. jirovecii* infection is often associated with T cell dysfunction. Rituximab, on the other hand, can affect CD4 + T cell production by affecting B cells, leading to an increased risk of *P. jirovecii* infection [51, 52]. Several therapeutic options exist for the treatment of PJP and TMP-SMX is the first-line agent and drug of choice [21, 43]. No agent has been shown to have better outcomes than TMP-SMX. TMP-SMX is also the drug of choice for PJP prophylaxis. Daily or thrice weekly dosing is equally effective [53]. Jiang et al. [54] showed in a meta-analysis that lymphoma patients who received rituximab chemotherapy had a significantly increased risk of PJP, but that preventive therapy was highly effective in preventing PJP. Recently, several studies indicated that prophylactic anti-*Pneumocystis* treatment with TMP-SMX decreased the incidence of IP in patients with B-cell lymphoma and receiving chemotherapy [9, 55]. These findings suggested that *P. jirovecii* infection plays an important role in the pathogenesis of chemotherapy-related IP. In addition, the clinical manifestations of PJP patients are non-specific, so rapid and accurate diagnosis of PJP patients is very important for clinical prognosis. A recent retrospective study showed that in non-HIV-infected patients, the sensitivity of mNGS to diagnose PJP reached 100%, and the specificity of mNGS (96.3%) significantly exceeded that of BDG [56]. In this study, the detection rate of mNGS was also high (12/15, 80%). Therefore, we believe that mNGS has good performance in diagnosing PJP.

PJP and drug-induced IP may have a similar imaging presentation of diffuse pulmonary interstitial infiltration. Several studies tried to describe the different characteristics of them. Diffuse GGO or diffuse interstitial infiltration was frequent in patients with PJP [11, 57, 58]; in contrast, more than one focal alveolar pattern was observed in about half the patients with rituximab-induced interstitial lung disease [59]. Most PJPs (95%) show diffuse interstitial infiltration with nonzonal predominance and PJP showed a significant trend toward more severe disease [57]. In our study, 86.7% (13/15) of patients showed diffuse GGO. And in patients with *P. jirovecii* detected, diffuse GGO was observed in 92.3% (12/13) of cases, which met the characteristics of PJP.

CMV infections were the most frequently reported in patients after allo-HSCT [60], but they are less common in patients after conventional chemotherapy. In our study, the detection rate of CMV was also high (4/15,26.7%), suggesting that more research in this area is warranted in the future.

Cytomegalovirus infection is the most common in patients after allogeneic hematopoietic stem cell transplantation [52], but less common in patients after conventional chemotherapy. In addition, the lungs of PJP patients were often accompanied by CMV infection [61, 62]. In our study, the detection rate of CMV was also high (4/15,26.7%), with co-infection of CMV and *P. jirovecii* detected in 3 patients. In addition, of the four CMV-positive patients, only two patients received ganciclovir antiviral therapy, and the other two patients who did not receive treatment eventually recovered. This may be because CMV-specific T cell responses are intact when CMV is reactivated, and the host may eventually control viral replication without developing CMV pneumonia [61, 63]. For non-immunocompromised patients, CMV infection is generally considered benign and self-limiting [64]. Previous studies have shown that anti-CMV treatment does not affect the mortality and severity of PJP in HIV-infected people with interstitial pneumonia [65]. Similar results were found in non-HIV-infected patients [61]. The 4 CMV positive patients in this study were all detected by mNGS, while the conventional test was negative, which indicates that the mNGS test is more sensitive. However, critical readings for NGS used as markers of PJP and CMV have not yet been determined, and early bronchoscopy and mNGS are recommended for immunocompromised patients.

We also found some discordance between mNGS versus culture results. *Klebsiella pneumoniae* and *Staphylococcus aureus* were respectively identified in P4 and P12 by culture of BALF, but not identified by mNGS. One possible reason may be the contamination during culture. Another possible reason may be associated with the false negative of mNGS. Several reports suggested that the sensitivity of mNGS is not superior to that of culture for recognizing common bacteria (excluding MTB and anaerobes) [38, 66]. Therefore, it is necessary to combine mNGS and traditional microbiological methods to identify clinical infection.

Of the 15 enrolled patients, 5 patients recovered after receiving antimicrobial treatments alone without using glucocorticoids, indicating that the cause of interstitial pneumonia in these patients may be opportunistic infections instead of lung damage. From our results, we confer that the previous view of drug-induced lung injury rather than infection being the main pathogenesis of IP after lymphoma chemotherapy (especially rituximab-based chemotherapy) is debatable. Instead, opportunistic infections, especially *P.jirovecii* infection is potentially the major factor in the pathogenesis of IP.

This study has certain shortcomings. Firstly, as a retrospective study, there were selection bias and recall bias which was inevitable. Secondly, the toluidine blue ‘O’ staining and PCR assay for *P.jirovecii* and the BALF-GM or BALF-BGD tests were not performed. Thirdly, the sample size of the enrolled population was small and controls were lacking. Finally, it was difficult to distinguish pathogens from colonization to infection due to the unbiased detection of mNGS without a unified standard. In the future, we will conduct prospective studies and further expand the sample size, and explore the feasibility of guiding treatment options in line with the mNGS results.

## Conclusion

In this study, we found that using mNGS test of BALF, the pathogen detection rate was much higher than that of traditional methods in lymphoma patients with chemotherapy-related IP. Notably, there was an especially high detection rate of *P. jirovecii*. We should be more alert to *P. jirovecii* infection in chemotherapy-related IP.

### Electronic supplementary material

Below is the link to the electronic supplementary material.

Supplementary Material 1

Supplementary Material 2

## Data Availability

All data generated or analyzed during this study are included in this published article.

## Abbreviations

IP: interstitial pneumonia
mNGS: metagenomic next-generation sequencing
BALF: bronchoalveolar lavage fluid
CMV: cytomegalovirus
EBV: Epstein-Barr virus
BDG: beta-D-glucan
GM: galactomannan
IPI: international prognostic index
CT: computed tomography
CRP: C-reactive protein
DLBCL: diffuse large B-cell lymphoma
MCL: mantle cell lymphoma
HL: Hodgkin’s lymphoma, FL:follicular lymphoma
ENKL: extranodal NK/T cell lymphoma
PTCL-NOS: peripheral T-cell lymphoma-NOS
allo-HSCT: allogeneic hematopoietic stem cell transplantation
GGO: ground-glass opacities
PJP: *Pneumocystis jirovecii* pneumonia
TMP-SMX: trimethoprim sulfamethoxazole
