# Supplementary material for: Pneumocystis jirovecii with high probability detected in bronchoalveolar lavage fluid of chemotherapy-related interstitial pneumonia in patients with lymphoma using metagenomic next-generation sequencing technology
Source: Infect Agent Cancer. 2023 Dec 6;18:80. doi: 10.1186/s13027-023-00556-1 (PMC10698987; doi:10.1186/s13027-023-00556-1)
Supplement: Supplementary file 1 — Supplementary Material 1 [file 13027_2023_556_MOESM1_ESM.docx]

**Table 1** Basic characteristics of the 15 patients with IP after chemotherapy

| Patient ID | Sex | Age (years) | Histology | IPI score | Smoking | Basic lung disease | Chemotherapy | Cycles of chemotherapy before IP |
| --- | --- | --- | --- | --- | --- | --- | --- | --- |
| P1 | male | 33 | DLBCL | 2 | yes | no | R-CHOP  R-CDOP | 4 |
| P2 | male | 57 | MCL | 2 | no | bronchiectasis | R-CHOP  CHOP | 3 |
| P3 | male | 54 | HL | 1 | yes | no | ABVD | 2 |
| P4 | male | 49 | DLBCL | 3 | yes | no | R-ECHOP  R-CDOP | 4 |
| P5 | female | 59 | DLBCL | 1 | no | no | R-CHOP | 3 |
| P6 | female | 62 | DLBCL | 1 | no | no | CHOP  R-CHOP | 4 |
| P7 | male | 61 | FL | 2 | no | no | R-CHOP | 4 |
| P8 | male | 54 | DLBCL | 2 | no | no | R-CDOP | 5 |
| P9 | female | 57 | DLBCL | 3 | no | no | R-CHOP  R-CDOP  R-ICE | 14 |
| P10 | male | 64 | DLBCL | 3 | yes | no | ECHOP  R-ECHOP | 3 |
| P11 | male | 55 | DLBCL | 1 | no | no | R-CDOP  R-CHOP | 8 |
| P12 | male | 30 | ENKL | 0 | no | no | P-GIDE  DHAP  allo-HSCT  Prednisone | 20 |
| P13 | female | 59 | PTCL-NOS | 2 | no | no | CDOP | 1 |
| P14 | male | 58 | DLBCL | 3 | no | no | R-EPOCH | 2 |
| P15 | male | 62 | DLBCL | 2 | no | no | RCDOP | 2 |

IPI: international prognostic index; DLBCL: diffuse large B-cell lymphoma; MCL: mantle cell lymphoma; HL: Hodgkin’s lymphoma, FL: follicular lymphoma; ENKL: extranodal NK/T cell lymphoma; PTCL-NOS: peripheral T-cell lymphoma-NOS; R: rituximab; CHOP: cyclophosphamide, epirubicin, vinorelbine, and dexamethasone/prednisone; CDOP: cyclophosphamide, doxorubicin hydrochloride liposome, vinorelbine, and dexamethasone/prednison; ECHOP: etoposide, cyclophosphamide, epirubicin, vinorelbine, and dexamethasone/prednison; EPOCH: cyclophosphamide, epirubicin, vinorelbine, etoposide, and dexamethasone; ABVD: doxorubicin hydrochloride liposome, bleomycin, vinorelbine, and dacarbazine; ICE: ifosfamide, etoposide and cisplatin; P-GIDE: pegaspargase plus gemcitabine, ifosfamide, dexamethasone, and etoposide; DHAP: cisplatin, cytarabine and dexamethasone; allo-HSCT: allogeneic hematopoietic stem cell transplantation.
