# Supplementary material for: Pneumocystis jirovecii with high probability detected in bronchoalveolar lavage fluid of chemotherapy-related interstitial pneumonia in patients with lymphoma using metagenomic next-generation sequencing technology
Source: Infect Agent Cancer. 2023 Dec 6;18:80. doi: 10.1186/s13027-023-00556-1 (PMC10698987; doi:10.1186/s13027-023-00556-1)
Supplement: Supplementary file 2 — Supplementary Material 2 [file 13027_2023_556_MOESM2_ESM.docx]

**Table 2** Clinical symptoms, CT findings, laboratory test results and microbial detection results of patients at the time of IP diagnosis

| Patient ID | Symptoms | CT findings | Neutrophil count (*10^9/L) | CRP  (mg/L) | BDG and GM assays | Routine laboratory staining and cultures | mNGS outcome | Course of prednisone  ≥20 mg/d | Antimicrobial therapy | Recovery time |
| --- | --- | --- | --- | --- | --- | --- | --- | --- | --- | --- |
| P1 | Fever  Dyspnea | Diffuse GGO | 10 | 98 | Negative | Negative | *Pneumocystis jirovecii* | 5 days | TMP-SMX, moxifloxacin | 5 days |
| P2 | Fever  Dyspnea | Diffuse GGO | 8.3 | 39.3 | Negative | Negative | *Campylobacter mucosa*  *Pneumocystis jirovecii* | no glucocorticoids were used | TMP-SMX, piperacillin-tazobactam, imipenem/cilastatin | 5 days |
| P3 | Fever | Diffuse GGO | 0.6 | 83 | Negative | Negative | *Pseudostreptococcus pneumoniae*  EBV  *Pneumocystis jirovecii* | no glucocorticoids were used | TMP-SMX, piperacillin-tazobactam | 20 days |
| P4 | Fever  Dyspnea | Diffuse patchy exudation | 13.3 | 62 | Negative | Klebsiella pneumoniae | EBV  *Candida albicans* | 3 months | Caspofungin, linezolid, imipenem/cilastatin | 3.5 months |
| P5 | Fever Dyspnea | Diffuse GGO | 2.2 | 10.6 | Positive BDG assay (361.9 pg/mL) | Negative | CMV  *Pneumocystis jirovecii* | 3 months | TMP-SMX, caspofungin, moxifloxacin, voriconazole | 1 year |
| P6 | Fever  Dyspnea | Diffuse patchy exudation | 0.6 | 61 | Negative | Negative | CMV  *Pneumocystis jirovecii* | 14 days | TMP-SMX, voriconazole, moxifloxacin, teicoplanin | 2 months |
| P7 | Fever  Dyspnea | Diffuse GGO | 2.2 | 14.1 | Negative | Negative | *Pneumocystis jirovecii* | 1.5 months | TMP-SMX, caspofungin, voriconazole | 5 months |
| P8 | Fever Dyspnea | Diffuse GGO | 1.1 | 277 | Positive BDG assay (275.34 pg/mL) | Negative | *Pneumocystis jirovecii* | 1 months | TMP-SMX, caspofungin, piperacillin-tazobactam | 1.5 months |
| P9 | Fever  Cough  Dyspnea | Diffuse GGO | 2.9 | 92.4 | Negative | Negative | EBV  *Pneumocystis jirovecii* | 3 months | TMP-SMX, caspofungin, piperacillin-tazobactam | 4.3 months |
| P10 | No symptom | Diffuse GGO | 2.4 | 25.2 | No data | Negative | Negative | 5 days | TMP-SMX | 9 days |
| P11 | Fever  Dyspnea | Diffuse GGO | 20.8 | 35.8 | No data | Negative | *Acinetobacter baumannii*  *Pneumocystis jirovecii* | 1 month | TMP-SMX, caspofungin, piperacillin-tazobactam | 15 days |
| P12 | Fever  Cough  Sputum | Diffuse GGO | 3 | 34 | Positive BDG assay (153.72 pg/mL) | Staphylococcus aureus | *Pseudomonas aeruginosa*  *Pneumocystis jirovecii* | no glucocorticoids were used | TMP-SMX, piperacillin-tazobactam | 30 days |
| P13 | Fever | Diffuse GGO | 10 | 11.8 | Negative | Negative | *Escherichia coli*  *Pneumocystis jirovecii*  CMV | no glucocorticoids were used | TMP-SMX, caspofungin, piperacillin-tazobactam, ganciclovir | 2 months |
| P14 | Fever | Diffuse GGO | 8.3 | 115 | Positive BDG assay  (129.14 pg/mL) | Negative | *Pneumocystis jirovecii* | no glucocorticoids were used | TMP-SMX, piperacillin-tazobactam | 15 days |
| P15 | Fever Dyspnea | Diffuse GGO | 0.6 | 60.6 | Negative | Negative | CMV | 15 days | TMP-SMX, piperacillin-tazobactam, ganciclovir | 1 month |

CT: computed tomography; GGO: ground-glass opacities; CRP: C-reactive protein; BDG: beta-D-glucan; GM: galactomannan; mNGS: metagenomic next-generation sequencing; CMV: Cytomegalovirus; EBV: Epstein-Barr virus; TMP-SMX: trimethoprim-sulfamethoxazole.

.
